# Dispensability of the SAC Depends on the Time Window Required by Aurora B to Ensure Chromosome Biorientation

**DOI:** 10.1371/journal.pone.0144972

**Published:** 2015-12-14

**Authors:** Marta Muñoz-Barrera, Isabel Aguilar, Fernando Monje-Casas

**Affiliations:** 1 CABIMER, Consejo Superior de Investigaciones Científicas (CSIC), Sevilla, Spain; 2 Departamento de Genética, Universidad de Sevilla, Sevilla, Spain; Florida State University, UNITED STATES

## Abstract

Aurora B and the spindle assembly checkpoint (SAC) collaborate to ensure the proper biorientation of chromosomes during mitosis. However, lack of Aurora B activity and inactivation of the SAC have a very different impact on chromosome segregation. This is most evident in *Saccharomyces cerevisiae*, since in this organism the lack of Aurora B is lethal and leads to severe aneuploidy problems, while the SAC is dispensable under normal growth conditions and mutants in this checkpoint do not show evident chromosome segregation defects. We demonstrate that the efficient repair of incorrect chromosome attachments by Aurora B during the initial stages of spindle assembly in budding yeast determines the lack of chromosome segregation defects in SAC mutants, and propose that the differential time window that Aurora B kinase requires to establish chromosome biorientation is the key factor that determines why some cells are more dependent on a functional SAC than others.

## Introduction

The correct distribution of the genetic material during mitosis requires the attachment of all chromosomes to spindle microtubules. A single unattached chromosome triggers the spindle assembly checkpoint (SAC), which halts cell cycle progression in metaphase through the inhibition of Cdc20, a cofactor of the anaphase-promoting complex/cyclosome (APC/C) [1, 2]. Additionally, cells must also ensure that each of the sister chromatids of the same chromosome attach to a different spindle pole, a state known as amphitelic attachment or chromosome biorientation. Aurora B kinase, the enzymatic component of the chromosome passenger complex (CPC), is crucial to achieve chromosome biorientation, and collaborates with the SAC to correct erroneous kinetochore-microtubule (KT-MT) attachments [3, 4]. A linear model has been proposed to explain the resolution of syntelic chromosomal attachments (when both sister chromatids bind microtubules emanating from the same spindle pole) by Aurora B and the SAC. According to this model, Aurora B senses incorrect attachments that originate a lack of tension in the spindle, and destabilizes these connections generating unattached kinetochores that trigger the SAC. Activation of the SAC then provides the cells with time to fix all the erroneous attachments before progressing into anaphase [4, 5]. It has been recently suggested that, in addition to the destabilization of kinetochore attachments, phosphorylation of Aurora B substrates in the kinetochore is important in preventing SAC silencing until chromosome bi-orientation is achieved [6]. Additionally, relocation of the CPC from centromeres to the spindle midzone is required later in anaphase in order to avoid reengagement of the SAC once that sister chromatids separate in anaphase and tension is lost again [7, 8]. Based on the previously described linear model for the resolution of syntelic attachments by Aurora B, it would be expected that both a deficiency in Aurora B or in the SAC led to similar chromosome segregation problems. However, the phenotypes of Aurora B and SAC mutants are very different in terms of chromosome segregation defects. This is most evident in *Saccharomyces cerevisiae*, since in this organism *IPL1* (the gene that encodes the Aurora B homolog) is an essential gene and its inactivation leads to severe chromosome missegregation [9], while SAC mutants are perfectly viable under normal growth conditions and do not show any evident chromosome segregation defects [10]. In this manuscript, we aim to reconcile the observations regarding the differential behavior of *ipl1* and SAC mutants with the currently accepted model for the resolution of syntelic attachments by the combined action of Aurora B and the SAC. Our results demonstrate that the dispensability of the SAC in *S*. *cerevisiae* is due to the efficient resolution of erroneous chromosomal attachments by Ipl1 during the initial stages of spindle assembly. Finally, we also propose that these results can be extrapolated to explain the differential dependence of cells from distinct organisms and different cell types within the same organism on a functional SAC for their viability.

## Results and Discussion

### Reduced Ipl1 activity and SAC deficiency lead to synergistic defects in chromosome segregation and cell viability

**Fig 1:**
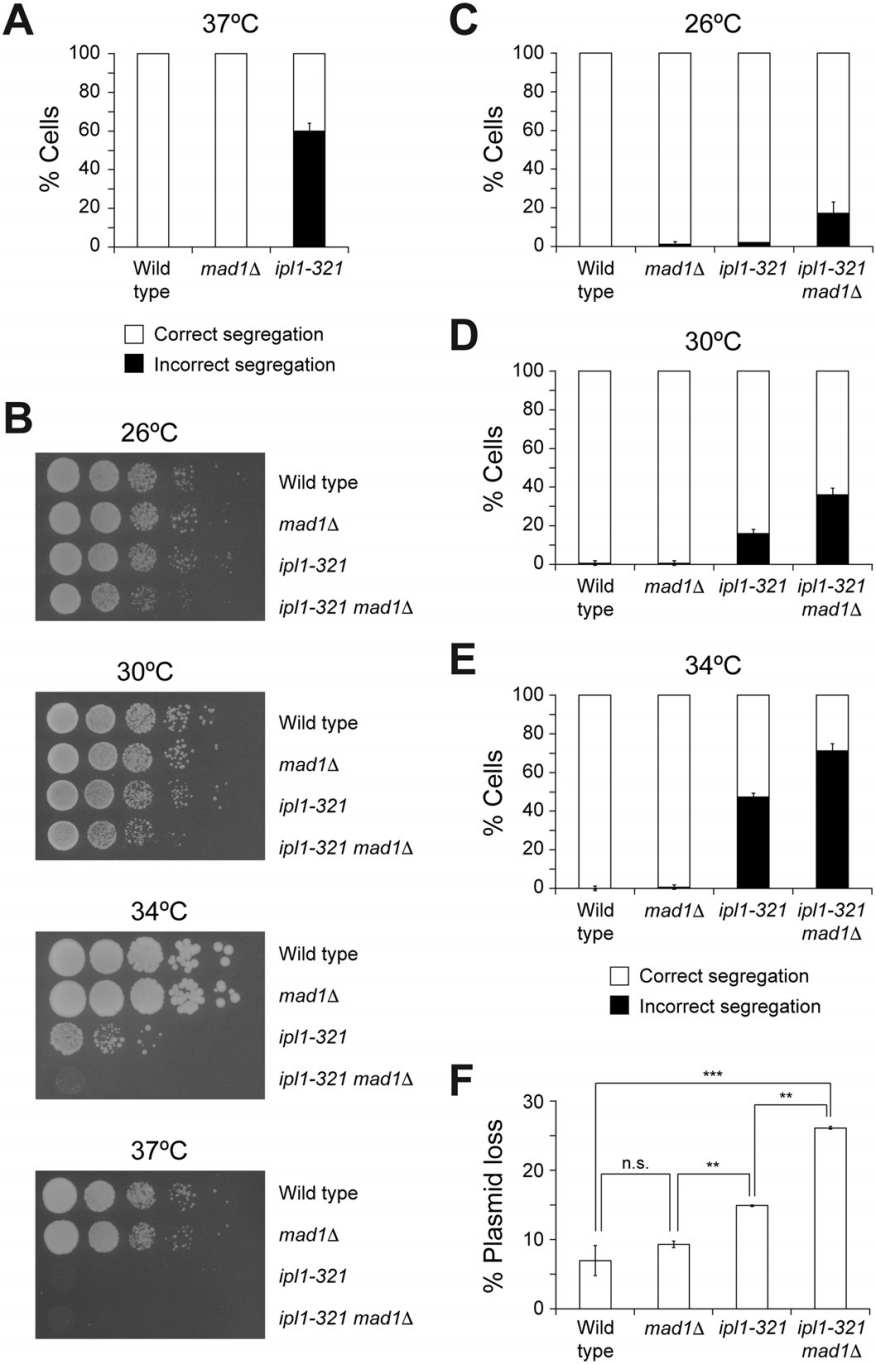
Synergistic defects associated to the lack of SAC and reduced Ipl1 activity. (A) Wild type (F955), *mad1*Δ (F142) and *ipl1-321* (F323) cells carrying CrIV-GFP were grown at 25°C in YPD, arrested in G1 with 5 μg/ml α-factor, and released at 37°C into medium without pheromone. Percentages of cells displaying correct (white bars) and incorrect (black bars) segregation of CrIV-GFP in anaphase are shown for each strain. Error bars indicate SD (n = 3). (B) 10-fold serial dilutions of wild type (F496), *mad1*Δ (F350), *ipl1-321* (F267), or *ipl1-321 mad1*Δ (F2414) liquid cultures grown in YPD at 25°C were spotted on YPD plates at the indicated temperatures to analyze cell viability. (C-E) Wild type (F955), *mad1*Δ (F142), *ipl1-321* (F323), or *ipl1-321 mad1*Δ (F2493) cells carrying CrIV-GFP were synchronized in G1 as in (A), and released into YPD medium without pheromone at the indicated temperatures. Percentages of cells displaying correct (white bars) and incorrect (black bars) segregation of CrIV-GFP in anaphase are shown for each strain. Error bars indicate SD (n = 3). (F) Plasmid-loss assays with wild type (F496), *mad1*Δ (F350), *ipl1-321* (F267), or *ipl1-321 mad1*Δ (F2414) cells carrying the pRS316 plasmid. The graph shows the median values and standard deviations of three fluctuation tests for every strain, each one performed from six independent colonies. Statistically significant (***, P < 0.001; and **, P < 0.01) or non-significant (n.s.) differences according to a two-tailed t-test are also shown.

Different hypotheses could be initially considered in order to explain the absence of chromosome segregation defects in SAC mutants during the normal growth of *S*. *cerevisiae*, in contrast to the dramatic phenotype shown by yeast cells lacking Ipl1 activity. As such, it has been suggested that Ipl1 could hold cell cycle progression independently of the SAC [11], which would ensure the time window that the kinase needs to fix all erroneous KT-MT attachments in the absence of the checkpoint. A two-hybrid interaction between Ipl1 and Apc1 (the major subunit of the APC/C) has been recently detected in a global interaction study [12]. Additionally, Aurora A (another member of the Aurora kinase family) has been shown to interact with Cdc20 in *Xenopus* egg extracts [13]. Thus, Ipl1 could directly inhibit the activity of APC/C^Cdc20^ in a SAC-independent manner. However, and despite several attempts to verify a putative physical interaction between Ipl1 and the APC/C or Cdc20 that could uncover a regulation of the metaphase to anaphase transition by Aurora B, we were unable to find any evidence of a direct association between these proteins. Therefore, we sought for an alternative hypothesis. The different phenotypes associated to a lack of Ipl1 activity and to defects in the SAC in budding yeast could be also explained if Ipl1 efficiently corrected all syntelic KT-MT attachments during the initial stages of spindle assembly under normal growth conditions, so that the SAC would be satisfied (or not even activated) before the cells are ready to enter the metaphase to anaphase transition. A prediction of this last hypothesis is that the viability of cells with reduced Ipl1 activity should be more dependent on a functional SAC. To test this prediction, we made use of the thermosensitive *ipl1-321* allele, which encodes a mutant Ipl1 protein that displays a ~12-fold reduction in its kinase activity [14]. Despite the presence of a functional SAC (as demonstrated, for example, by the fact that *ipl1-321* cells arrest in metaphase in the presence of drugs that depolymerize the spindle microtubules [11]), an *ipl1-321* mutant displayed severe chromosome segregation defects during anaphase at the restrictive temperature, which were evidenced by the presence of both sister chromatids of a GFP-tagged chromosome IV (CrIV-GFP) [15] either in the mother or in the daughter cell (Fig 1A and Figure A in S1 File). Remarkably, and in agreement with our prediction, the viability of *ipl1-321* cells was reduced at the permissive and semi-permissive temperatures when they additionally carried a deletion of the *MAD1* gene, which encodes an essential SAC component (Fig 1B). This cell viability defect was associated to an increase in chromosome missegregation (Fig 1C, 1D and 1E and Figure A in S1 File). Accordingly, increased genomic instability with respect to the *ipl1-321* mutant was also evident by analyzing the rate of plasmid loss in *ipl1-321 mad1*Δ cells (Fig 1F). Similar synergistic viability defects were observed when Ipl1 activity was reduced in *mad1*Δ cells using the ATP analogue-sensitive Ipl1-as5 kinase [5] (Figure B in S1 File). Taken together, our results show that the viability of cells with compromised Ipl1 activity (*e*.*g*., *ipl1-321* cells at the semi-permissive temperatures) is further impaired in the absence of a functional SAC, and demonstrate that simultaneous inactivation of Ipl1 and the SAC leads to synergistic defects during chromosome segregation.

### A new conditional allele to delay activation of Ipl1 during the cell cycle

**Fig 2:**
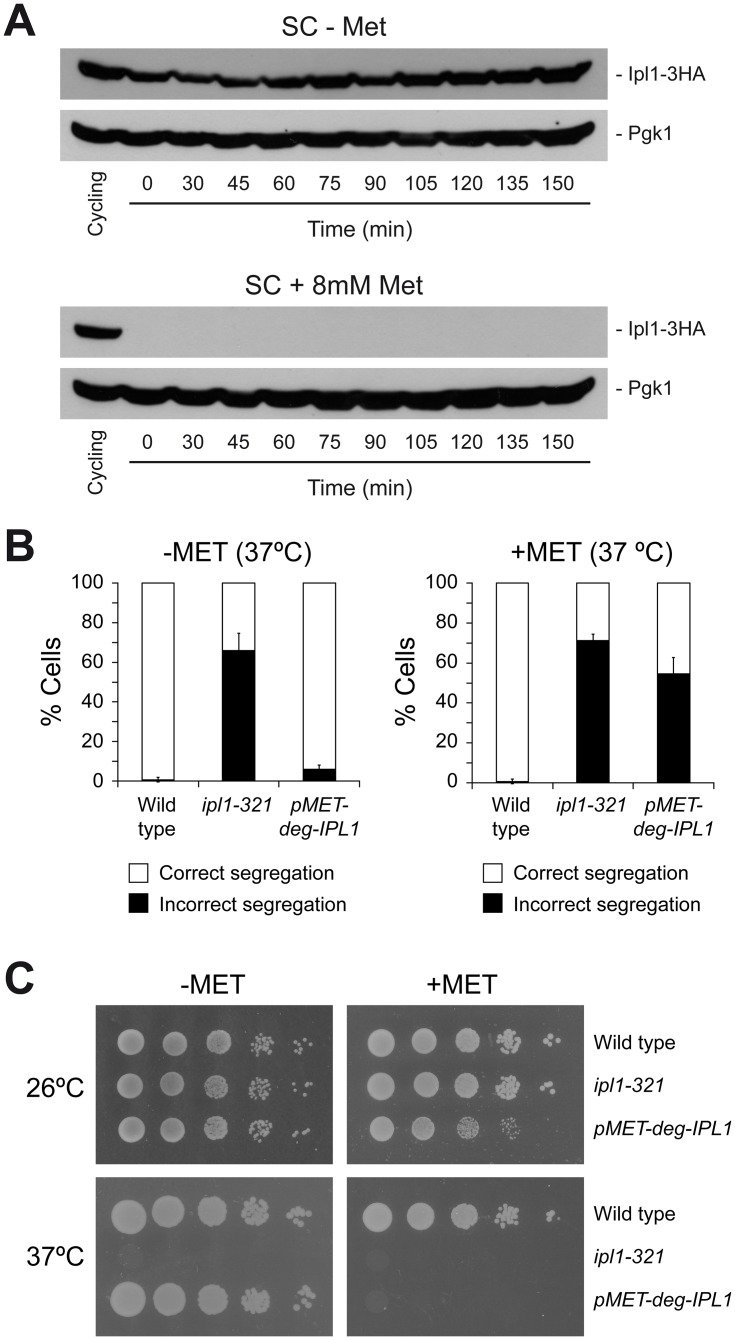
A new *IPL1* conditional allele. (A-B) Cells were grown at 25°C in SC medium without methionine (SC-MET), arrested in G1 with 5 μg/ml α-factor, and released at 37°C into SC medium containing (+MET) or not (-MET) 8 mM methionine and without pheromone. (A) Western blots show Ipl1-3HA levels at the indicated time points and in a cycling culture of *pMET3-Ub-DHFR-IPL1-3HA* (F1595) cells. Pgk1 was used as loading control. (B) Percentages of wild type (F955), *ipl1-321* (F323), or *pMET3-Ub-DHFR-IPL1* (F1517) cells displaying correct (white bars) and incorrect (black bars) segregation of CrIV-GFP in anaphase. Error bars indicate SD (n = 3). (C) 10-fold serial dilutions of wild type (F496), *ipl1-321* (F323), or *pMET3-Ub-DHFR-IPL1* (F1124) liquid cultures grown in SC-MET at 25°C were spotted on SC-MET and SC+MET plates at the indicated temperatures to analyze cell viability.

We next reasoned that a delay in the onset of Ipl1 activity during cell cycle progression would generate an artificial situation in which this kinase had a reduced time window to ensure chromosome biorientation and that, in this scenario, the SAC should become critical to halt cell cycle progression and allow the Aurora B homolog to fix erroneous KT-MT attachments before the cells activate APC/C^Cdc20^ and commit to anaphase. This experimental approach required a conditional *IPL1* allele to precisely control the expression of this kinase. Since *ipl1-321 mad1*Δ cells displayed chromosome segregation defects already at the permissive temperature (Fig 1C), we generated a strain in which the sole copy of *IPL1* was under the control of the methionine-repressible *MET3* gene promoter, and additionally fused to a temperature-inducible degron module that allows for a fast degradation of the protein at 37°C [16]. Cells carrying the *pMET3-Ub-DHFR-IPL1* allele could not express Ipl1 in methionine-containing media at 37°C, which led to severe chromosome segregation defects (similar to *ipl1-321* cells) and to lethality (Fig 2A, 2B and 2C). However, cells grew as the wild type in medium without methionine at the permissive, and even at the restrictive temperatures (Fig 2B and 2C).

**Fig 3:**
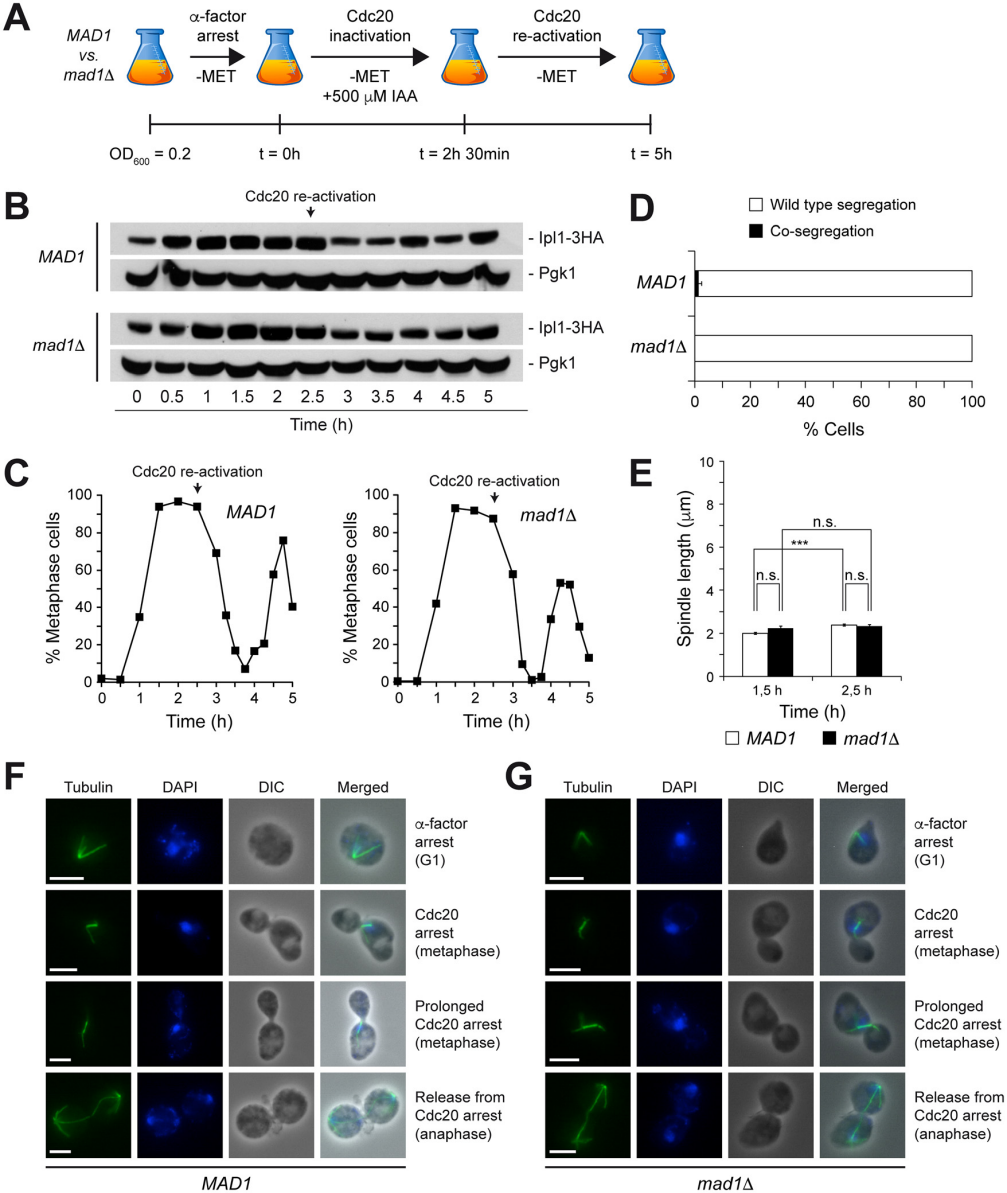
Functionality of the *pMET3-Ub-DHFR-IPL1* allele. (A-F) *pMET3-Ub-DHFR-IPL1-3HA CDC20-AID* (*MAD1*; F1940) and *pMET3-Ub-DHFR-IPL1-3HA CDC20-AID mad1*Δ (*mad1*Δ; F1942) cells carrying CrIV-GFP were grown at 25°C in SC-MET, arrested in G1 with 5 μg/ml α-factor, and released at 25°C into SC medium without pheromone or methionine but containing 500 μM IAA. After 2.5 h, cells were washed and resuspended in SC at 25°C without methionine or IAA. (A) Scheme summarizing the experiment. (B) Western blots show Ipl1-3HA levels at the indicated time points. Pgk1 was used as loading control. (C) Cell cycle progression was analyzed by spindle (tubulin) and nuclear (DAPI) morphology. Percentages of metaphase cells are shown for each time point. (D) Percentages of cells displaying correct segregation (white bars) or co-segregation (black bars) of CrIV-GFP sister chromatids in anaphase. Error bars indicate SD (n = 3). (E) Average spindle length at the indicated time points. Error bars indicate SEM (n = 75). Statistically significant (***, P < 0.001) or non-significant (n.s.) differences according to a Student-Newman-Keuls multiple comparison test are also shown. (F-G) Representative images of tubulin (green) and DAPI (blue) in *pMET3-Ub-DHFR-IPL1-3HA CDC20-AID* (E; F1940) and *pMET3-Ub-DHFR-IPL1-3HA CDC20-AID mad1*Δ (F; F1942). DIC and merged images are also shown. Scale bar = 5 μm.

We next carried out an initial control experiment to test the ability of the *pMET3-Ub-DHFR-IPL1* allele to correct erroneous KT-MT attachments at the permissive temperature in the absence of a functional checkpoint. Cells also carried a *CDC20-AID* allele, which encodes an auxin-inducible degron [17] of Cdc20 that allows for its reversible inactivation by adding indole-3-acetic acid (IAA; a natural auxin) to the medium (Figure A in S2 File and Figure B in S2 File). After an initial G1 arrest, *pMET3-Ub-DHFR-IPL1 CDC20-AID* cells were allowed to synchronously enter the cell cycle in medium lacking methionine but with IAA, so that Ipl1 was expressed but Cdc20 was degraded (Fig 3A and 3B). Depletion of Cdc20 forced cells to arrest in metaphase due to APC/C inactivation (Fig 3C). Finally, Cdc20 was re-activated to allow cells to enter anaphase, and chromosome segregation was analyzed. After Cdc20 activation there seemed to be a slight decrease in the levels of Ipl1 protein, which could reflect an APC/C-dependent active degradation of Aurora B kinase, as previously suggested [18, 19]. In any case, this does not have any effect on protein function. Both CrIV-GFP sister chromatids were equally distributed between the mother and the daughter cells (Fig 3D), which confirmed that the *pMET3-Ub-DHFR-IPL1* allele was fully functional at the permissive temperature. Importantly, continuous expression of this allele ensured chromosome biorientation both in cells with a functional SAC and in *mad1*Δ cells (Fig 3D). This was further demonstrated by analyzing spindle morphology. During the Cdc20-dependent arrest, and both in the presence and in the absence of a functional SAC, *pMET3-Ub-DHFR-IPL1 CDC20-AID* cells exhibited a typical short metaphase spindle (~2 μm length), which was maintained until Cdc20 was reactivated and cells were allowed to enter anaphase (Fig 3E, 3F and 3G). This morphology indicated the existence of tension in the spindle as a consequence of the correct biorientation of the chromosomes, and therefore an efficient Ipl1 activity.

### A delayed activation of Ipl1 makes the SAC essential for proper chromosome segregation

**Fig 4:**
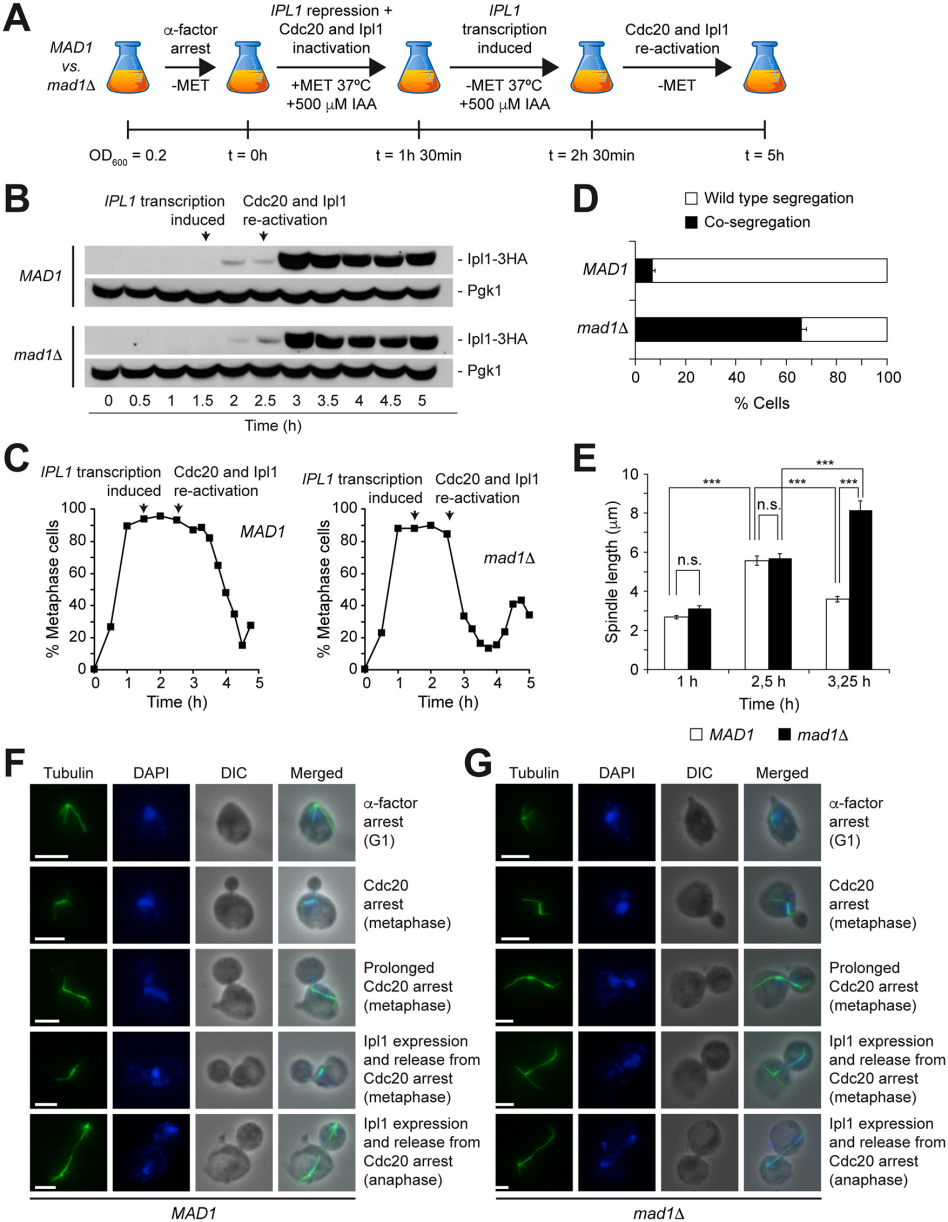
Delaying the onset of Ipl1 activity during cell cycle progression. (A-F) *pMET3-Ub-DHFR-IPL1-3HA CDC20-AID* (*MAD1*; F1940) and *pMET3-Ub-DHFR-IPL1-3HA CDC20-AID mad1*Δ (*mad1*Δ; F1942) cells carrying CrIV-GFP were synchronized in G1 in SC-MET as in Fig 3, and released at 37°C into SC medium without pheromone, but containing 8 mM methionine and 500 μM IAA. After 1.5 h, cells were transferred to medium without methionine but with 500 μM IAA, and also at 37°C. Finally, 1 h later, cells were washed and resuspended in SC at 25°C without methionine or IAA. (A) Scheme summarizing the experiment. (B) Western blots show Ipl1-3HA levels at the indicated time points. Pgk1 was used as loading control. (C) Cell cycle progression was analyzed by spindle (tubulin) and nuclear (DAPI) morphology. Percentages of metaphase cells are shown for each time point. (D) Percentages of cells displaying correct segregation (white bars) or co-segregation (black bars) of CrIV-GFP sister chromatids in anaphase. Error bars indicate SD (n = 3). (E) Average spindle length at the indicated time points. Error bars indicate SEM (n = 75). Statistically significant (***, P < 0.001) or non-significant (n.s.) differences according to a Student-Newman-Keuls multiple comparison test are also shown. (F-G) Representative images of tubulin (green) and DAPI (blue) in *pMET3-Ub-DHFR-IPL1-3HA CDC20-AID* (E; F1940) and *pMET3-Ub-DHFR-IPL1-3HA CDC20-AID mad1*Δ (F; F1942). DIC and merged images are also shown. Scale bar = 5 μm.

**Fig 5:**
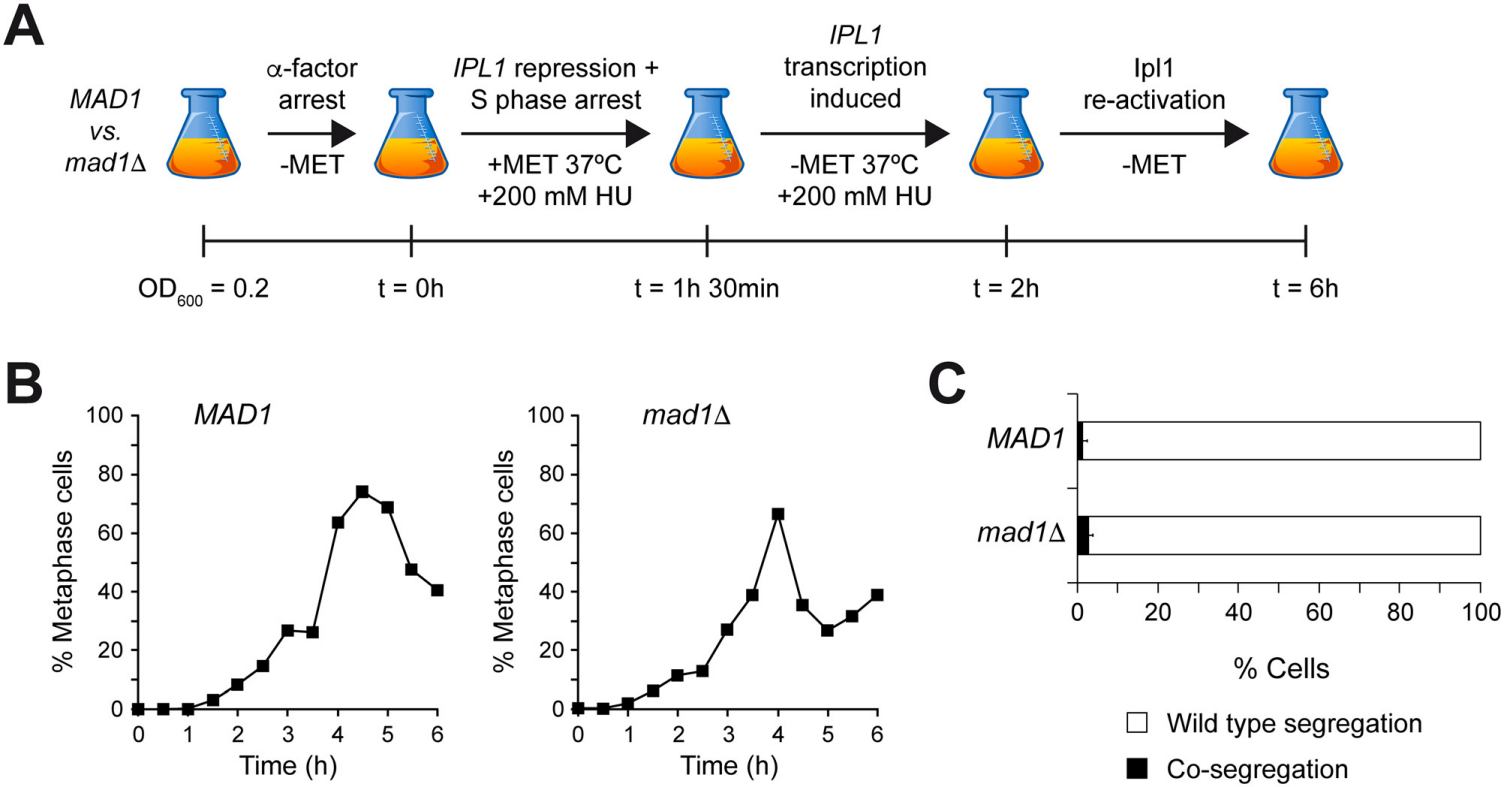
Delaying the onset of Ipl1 activity until S phase. (A-C) *pMET3-Ub-DHFR-IPL1-3HA CDC20-AID* (*MAD1*; F1940) and *pMET3-Ub-DHFR-IPL1-3HA CDC20-AID mad1*Δ (*mad1*Δ; F1942) cells carrying CrIV-GFP were grown at 25°C in SC medium without methionine, arrested in G1 with 5 μg/ml α-factor, and released into SC medium without pheromone but containing 8 mM methionine and 200 mM HU, and at 37°C. After 1.5 h, cells were transferred to medium without methionine but with 200 mM HU and at 37°C. Finally, 30 min later, cells were washed and resuspended in SC without methionine or HU, and at 25°C. (A) Scheme summarizing the experiment. (B) Cell cycle progression was analyzed by spindle (tubulin) and nuclear (DAPI) morphology. Percentages of metaphase cells are shown for each time point. (C) Percentages of cells displaying correct segregation (white bars) or co-segregation (black bars) of CrIV-GFP sister chromatids in anaphase. Error bars indicate SD (n = 3).

We finally used the conditional *pMET3-Ub-DHFR-IPL1* allele to delay the onset of Ipl1 activity during cell cycle progression and verify whether, in this situation, the ability of the cells to correct syntelic KT-MT attachments was dependent on the SAC. To this end, *pMET3-Ub-DHFR-IPL1 CDC20-AID* cells were again arrested in G1, but this time the cells were allowed to synchronously enter the cell cycle in conditions that repressed both Ipl1 and Cdc20 expression (Fig 4A and 4B). As cells entered the metaphase arrest caused by Cdc20 inactivation, *IPL1* transcription was initiated by removing methionine from the medium. Finally, once that the arrest was completed, expression of both Ipl1 and Cdc20 was allowed by placing cells at the permissive temperature and removing IAA, respectively (Fig 4A, 4B and 4C). *pMET3-Ub-DHFR-IPL1 CDC20-AID* cells temporally prolonged the metaphase arrest after Cdc20 expression was induced, but finally entered anaphase and exited mitosis (Fig 4C). During the metaphase arrest caused by Cdc20 depletion, the spindle abnormally elongated, which indicated that the lack of Ipl1 activity during the early stages of spindle assembly caused an accumulation of mono-oriented chromosomal attachments and, as a consequence, a reduction of the tension in the spindle (Fig 4E and 4F). However, the induction of Ipl1 expression allowed the repair of all erroneous KT-MT attachments and the re-establishment of chromosome biorientation, as evidenced by the recovery of the short metaphase spindle morphology (Fig 4E and 4F). Importantly, the metaphase arrest was maintained until chromosome biorientation was achieved, despite the induction of Cdc20 expression. Accordingly, both CrIV-GFP sister chromatids were equally distributed between the mother and the daughter cells in anaphase (Fig 4D). Strikingly, and in sharp contrast to cells with a functional SAC, *pMET3-Ub-DHFR-IPL1 CDC20-AID mad1*Δ cells immediately progressed into anaphase after Cdc20 expression, and did not temporally hold the metaphase arrest (Fig 4C). Additionally, both CrIV-GFP sister chromatids migrated to the same spindle pole in most cases, which indicated that Ipl1 was not able to repair syntelic attachments without the additional help from the SAC (Fig 4D). This was further demonstrated by the analysis of the spindle morphology. Again, delayed expression of Ipl1 and the subsequent accumulation of mono-oriented chromosomes determined an abnormal elongation of the spindle during the metaphase arrest. However, cells lacking a functional SAC never recovered the short metaphase spindle morphology after Ipl1 and Cdc20 expression, and proceeded to anaphase without repairing the syntelic KT-MT attachments (Fig 4E and 4G). Importantly, these results further argue against the ability of Ipl1 to induce a mitotic arrest independently of the SAC. Our results thus demonstrate that the SAC becomes essential for the efficient resolution of erroneous KT-MT attachments when the onset of Ipl1 activity is delayed and the kinase has an insufficient time window to ensure chromosome biorientation. In agreement with our model, no chromosome segregation defects were observed in *pMET3-Ub-DHFR-IPL1 CDC20-AID mad1*Δ cells when the experiment was repeated but delaying the onset of Ipl1 activity only until S phase, and therefore providing the kinase again with a wider time frame to correct syntelic chromosomal attachments (Fig 5A, 5B and 5C).

### Conclusions

Our results demonstrate that the efficient resolution of erroneous KT-MT attachments by Ipl1 during the initial stages of spindle assembly determines the differential phenotypes of Ipl1 and SAC mutants in budding yeast, as well as the dispensability of the checkpoint during the normal growth of this organism. Indeed, we have demonstrated that cells with reduced Ipl1 activity become more dependent on the SAC, which provides the hypo-functional kinase with enough time as to repair incorrect KT-MT attachments and prevent chromosome segregation defects. The same is true when the onset of Ipl1 activity is delayed during cell cycle progression. Hence, and in the absence of additional chromosomal attachment defects, the SAC is normally a safeguard mechanism that it is only necessary to provide Aurora B with an appropriate time window to ensure chromosome biorientation.

In contrast to *S*. *cerevisiae*, the SAC is essential for the viability of *C*. *elegans*, *Drosophila*, mouse and human cells [20–23]. However, cells lacking Aurora B activity and cells deficient in the SAC have also been shown to display different phenotypes in these organisms. In mouse models, simultaneous inactivation of both Aurora B and C, as well as homozygous mutations of INCENP, survivin or borealin (the other CPC components), lead to very early embryonic lethality (even at the 2-cell stage), with cells showing severe chromosome segregation defects [24–27]. In sharp contrast, and although a homozygous Mad2-null mutation also leads to lethality during embryogenesis, embryonic mouse cells lacking a functional SAC seem to grow normally until E5.5 [21]. Furthermore, lethality in these cells is associated to an increase in apoptosis, and mouse embryos null for both Mad2 and p53 are viable in culture [28], while p53 inactivation does not suppress the cell proliferation defects and apoptosis due to the lack of CPC activity [27]. Remarkably, it is only after gastrulation initiates (E6.5) that the SAC becomes essential during mouse embryogenesis. Gastrulation is an especially active period during which embryonic cells carry out a series of very rapid divisions [29, 30]. This would obviously provide Aurora B with less time to repair wrong KT-MT attachments, which would subsequently make cells to become more dependent on a functional SAC.

Finally, our results also help us to provide an answer to a long-standing question: why some cells are more dependent on a functional SAC for their viability than others. There are several differences with respect to the chromosome segregation process in budding yeast and higher eukaryotes. As such, while yeast chromosomes are attached to microtubules throughout most of the cell cycle, the chromosomes of higher eukaryotes bind to microtubules emanating from the spindle poles only during mitosis [31]. Additionally, and while only one microtubule attaches per kinetochore in budding yeast, the kinetochores from higher eukaryotes are simultaneously bound by several spindle microtubules [32]. These and other factors could influence the differential dependence on a functional SAC for cell viability. The later kinetochore capture by spindle microtubules is initiated and the greater the number of chromosomes and microtubules that attach per kinetochore, the more difficult it would be for Aurora B to ensure chromosome biorientation, and thus the more dependent that cells will become on the SAC to provide Aurora B with the time needed to repair all erroneous chromosome attachments. Furthermore, the same rationale could be used to explain why even different cells from the same organism display a distinct dependence on the SAC. In a mouse model, it has been shown that SAC deficiency is incompatible with the survival of hair follicle stem cells, while a lack of this checkpoint is well tolerated in cells from the interfollicular epidermis [33]. Interestingly, cells from the interfollicular epidermis spend more time in prometaphase than the hair follicle stem cells, which provides Aurora B with more time to ensure chromosome biorientation. We propose that it is the differential time window required for Aurora B to establish chromosome biorientation that determines the dispensability of the SAC for cell viability.

## Materials and Methods

### Strains

All strains are derivatives of W303 and are described in S1 Table.

### Plasmid loss assays

Plasmid loss assays were carried out as described in [34]. In short, cells carrying the centromeric pRS316 plasmid were grown on SC medium for 23 generations, after which they were diluted, plated on SC plates (to determine the total number of cells), and subsequently replicated on SC without uracil (in order to score for the number of cells losing the plasmid).

### Immunofluorescence microscopy

Immunofluorescence was carried out as described in [35]. Anti-tubulin (Abcam) and anti–rat FITC (Jackson ImmunoResearch) antibodies were used at 1:250. Microscope preparations were imaged using a DM6000 microscope (Leica) equipped with a 100x/1.40 NA oil immersion objective lens, A4, L5, and TX2 filters, and a DF350 digital charge-coupled device camera (Leica). Pictures were processed with LAS AF (Leica) and ImageJ (http://rsbweb.nih.gov/ij/) software.

### DAPI staining

Samples were fixed for 15min at RT in 2.5% formaldehyde, washed twice with 0.1M potassium phosphate buffer (pH 6.6), and resuspended in 0.1M potassium phosphate buffer (pH 7.4). Cells were first fixed for 10 min in 80% ethanol and then resuspended in 10 μg/ml DAPI. Microscope preparations were imaged as for immunofluorescence.

### Western blot analysis

Protein extracts were prepared as described in [36]. Ipl1-3HA was detected using HA.11 antibody (Covance) at 1:5000, and Pgk1 levels were measured using anti-Pgk1 antibody (Invitrogen) at 1:20000. Finally, anti–mouse HRP-linked antibody (GE Healthcare) was used at 1:10000. Protein signal was detected using the Western Bright ECL system (Advansta).

## Supporting Information

S1 File Reduced Ipl1 activity and SAC deficiency lead to synergistic defects in cell viability. (**Figure A**) Representative images of wild type (F955), *mad1*Δ (F142), *ipl1-321* (F323), or *ipl1-321 mad1*Δ (F2493) anaphase cells carrying CrIV-GFP and displaying correct or incorrect segregation of the GFP-tagged chromosome (CrIV-GFP, green). The segregated DNA masses (DIC, blue), as well as DIC and merged images are also shown. Scale bar = 5 μm. (**Figure B**) Wild type (F496), *mad1*Δ (F350), *ipl1-as5* (F1696), or *ipl1-as5 mad1*Δ (F1956) cells were grown in YPD at 25°C. Cell viability was determined by spotting 10-fold serial dilutions of the previous cultures onto YPD plates with or without 50 μM 3MB-PP1, an inhibitory ATP analogue, which were then incubated at 25°C. Note that DMSO was added to the control plates since the 3MB-PP1 stock solution was prepared in this solvent.
(TIF)

S2 File Characterization of the *CDC20-AID* allele. (**Figure A**) Wild type (F496), *pADH1-OsTir1* (F1664), and *pADH1-OsTir1 CDC20-AID* (F1704) cells were grown in YPD at 25°C. Cell viability was determined by spotting 10-fold serial dilutions of the previous cultures onto YPD plates containing (+500 μM IAA) or not (+EtOH) 500 μM IAA, which were then incubated at 25°C. IAA binding to TIR1 promotes the interaction between the E3 ubiquitin ligase complex SCF-TIR1 and the auxin-inducible degron, which induces degradation of the degron-tagged target protein. Note that EtOH was added to the control plates since the IAA stock solution was prepared in this solvent. (**Figure B**) *pADH1-OsTir1* cells carrying the *CDC20-AID* allele (F1704) were grown in YPD at 25°C, arrested in G1 with 5 μg/ml α-factor, and released into fresh medium with (+500 μM IAA) or without (+EtOH) 500 μM IAA and at 37°C. Cell cycle progression was analyzed by spindle (tubulin) and nuclear morphologhy (DAPI). Percentages of metaphase cells are shown for each time point.
(TIF)

S1 Table Strains. All strains are W303 derivatives. Only relevant differences in the genotype with respect to the wild type strain (F496) are shown in each case.
(DOCX)
