# Supplementary figures and images for: Dispensability of the SAC Depends on the Time Window Required by Aurora B to Ensure Chromosome Biorientation
Source: PLoS One. 2015 Dec 14;10(12):e0144972. doi: 10.1371/journal.pone.0144972 (PMC4682840; doi:10.1371/journal.pone.0144972)

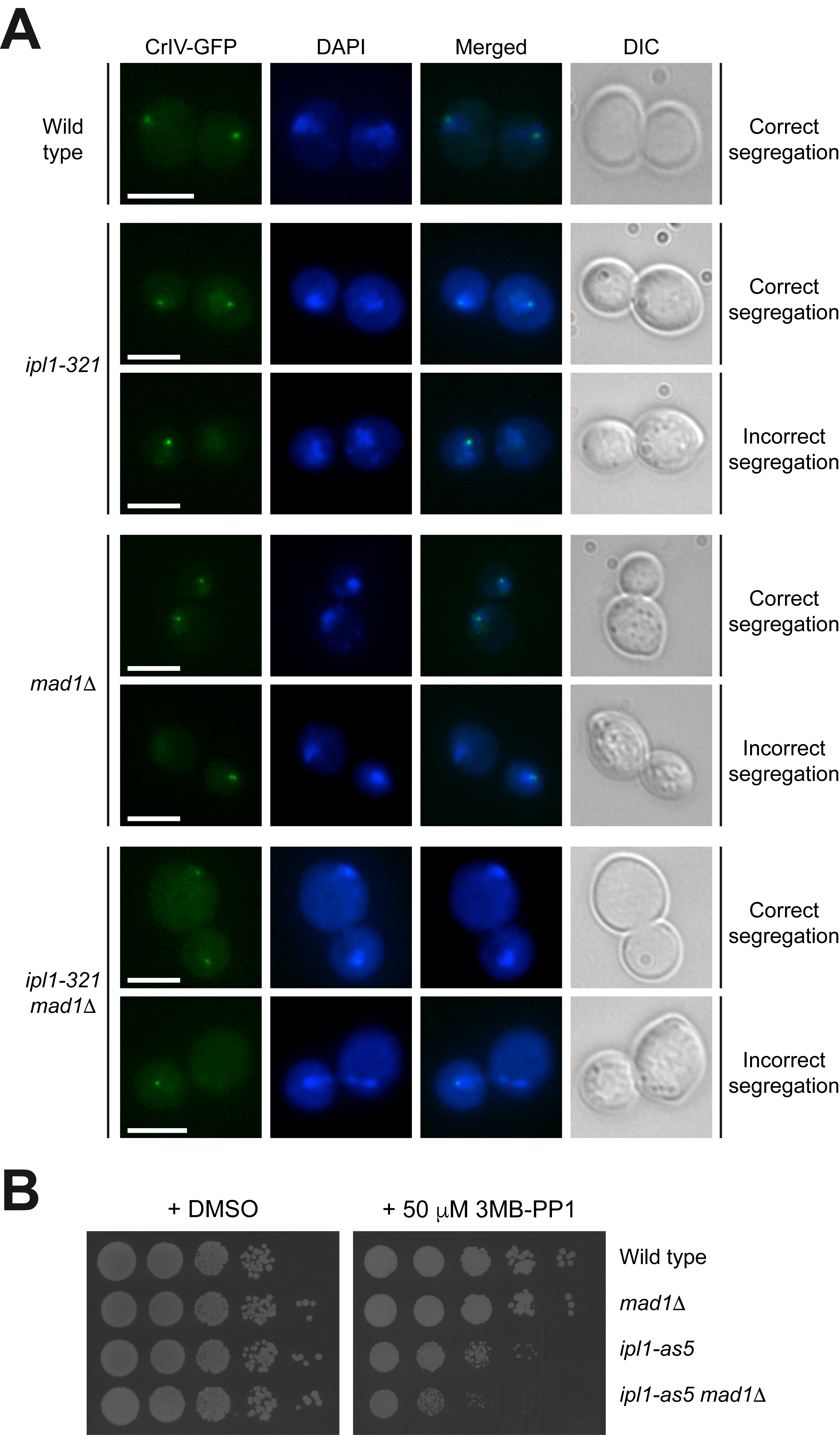

Supplement: S1 File — (Figure A) Representative images of wild type (F955), mad1Δ (F142), ipl1-321 (F323), or ipl1-321 mad1Δ (F2493) anaphase cells carrying CrIV-GFP and displaying correct or incorrect segregation of the GFP-tagged chromosome (CrIV-GFP, green). The segregated DNA masses (DIC, blue), as well as DIC and merged images are also shown. Scale bar = 5 μm. (Figure B) Wild type (F496), mad1Δ (F350), ipl1-as5 (F1696), or ipl1-as5 mad1Δ (F1956) cells were grown in YPD at 25°C. Cell viability was determined by spotting 10-fold serial dilutions of the previous cultures onto YPD plates with or without 50 μM 3MB-PP1, an inhibitory ATP analogue, which were then incubated at 25°C. Note that DMSO was added to the control plates since the 3MB-PP1 stock solution was prepared in this solvent. (TIF) [file pone.0144972.s001.tif]

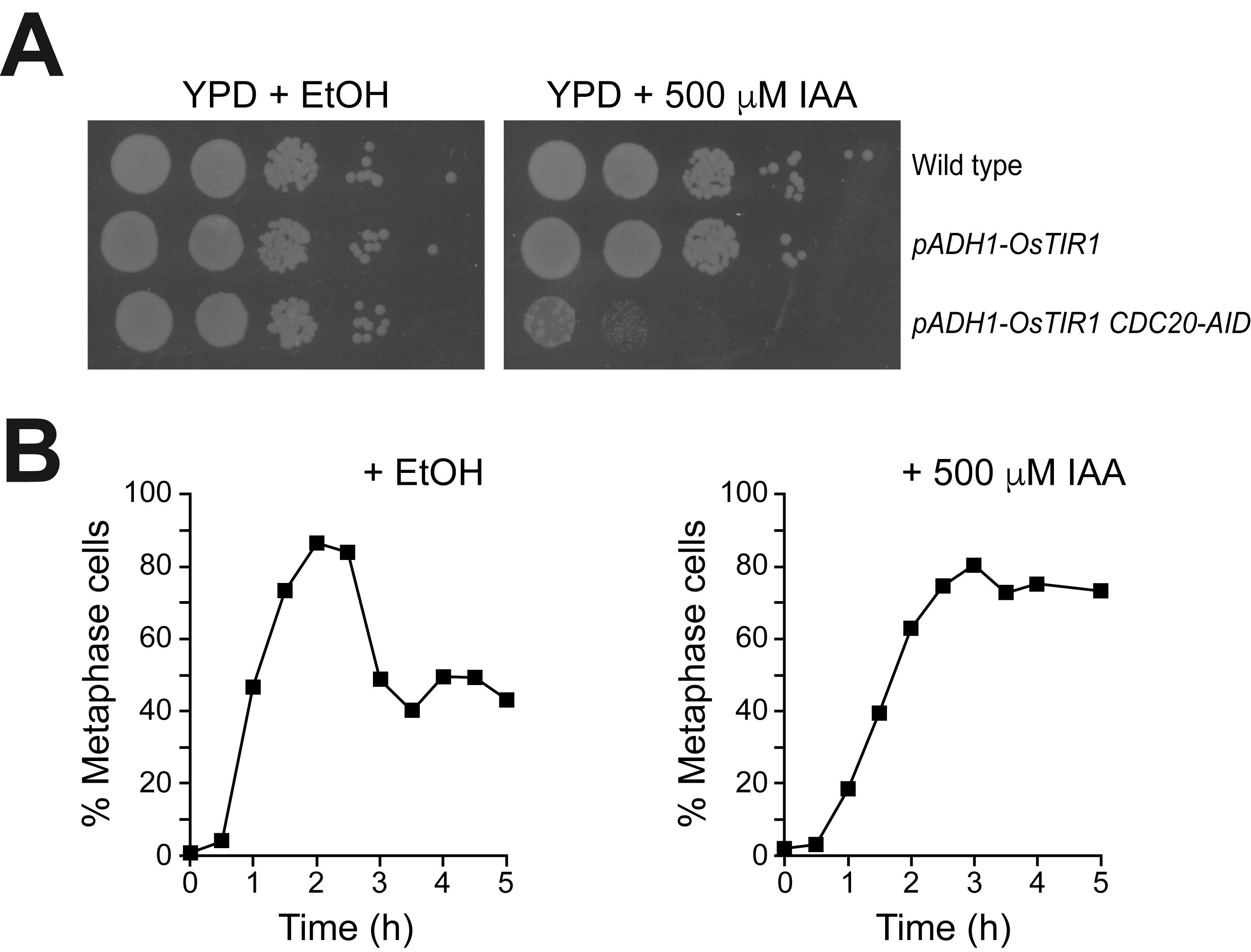

Supplement: S2 File — (Figure A) Wild type (F496), pADH1-OsTir1 (F1664), and pADH1-OsTir1 CDC20-AID (F1704) cells were grown in YPD at 25°C. Cell viability was determined by spotting 10-fold serial dilutions of the previous cultures onto YPD plates containing (+500 μM IAA) or not (+EtOH) 500 μM IAA, which were then incubated at 25°C. IAA binding to TIR1 promotes the interaction between the E3 ubiquitin ligase complex SCF-TIR1 and the auxin-inducible degron, which induces degradation of the degron-tagged target protein. Note that EtOH was added to the control plates since the IAA stock solution was prepared in this solvent. (Figure B) pADH1-OsTir1 cells carrying the CDC20-AID allele (F1704) were grown in YPD at 25°C, arrested in G1 with 5 μg/ml α-factor, and released into fresh medium with (+500 μM IAA) or without (+EtOH) 500 μM IAA and at 37°C. Cell cycle progression was analyzed by spindle (tubulin) and nuclear morphologhy (DAPI). Percentages of metaphase cells are shown for each time point. (TIF) [file pone.0144972.s002.tif]
