# Supplementary material for: Dispensability of the SAC Depends on the Time Window Required by Aurora B to Ensure Chromosome Biorientation
Source: PLoS One. 2015 Dec 14;10(12):e0144972. doi: 10.1371/journal.pone.0144972 (PMC4682840; doi:10.1371/journal.pone.0144972)
Supplement: S1 Table — All strains are W303 derivatives. Only relevant differences in the genotype with respect to the wild type strain (F496) are shown in each case. (DOCX) [file pone.0144972.s003.docx]

**S1 Table. Strains.**

All strains are W303 derivatives. Only relevant differences in the genotype with respect to the wild type strain (F496) are shown in each case.

| Strain | Relevant genotype |
| --- | --- |
| F496 | MATa wild type |
| F142 | MATa, *pURA3::tetR::GFP::LEU2*, *cenIV::tetOx448::URA3*, *mad1::HIS3MX6* |
| F267 | MATa, *ipl1-321* |
| F323 | MATa, *pURA3::tetR::GFP::LEU2*, *cenIV::tetOx448::URA3*, *ipl1-321* |
| F350 | MATa, *mad1::HIS3MX6* |
| F955 | MATa, *pURA3::tetR::GFP::LEU2*, *cenIV::tetOx448::URA3* |
| F1124 | MATa, *pIPL1::TRP1::pMET3-UB-DHFR-IPL1* |
| F1517 | MATa, *pIPL1::TRP1::pMET3-UB-DHFR-IPL1*, *pURA3::tetR::GFP::LEU2*, *cenIV::tetOx448::URA3* |
| F1595 | MATa, *pIPL1::TRP1::pMET3-UB-DHFR-IPL1-3HA::HIS3MX6* |
| F1664 | MATa, *ura3-1::ADH1-OsTIR1-9Myc::URA3* |
| F1696 | MATa, *ipl1-as5::HIS3::KanMX6*, *pURA3::tetR::GFP::LEU2*, *cenIV::tetOx448::URA3* |
| F1704 | MATa, *ura3-1::ADH1-OsTIR1-9Myc::URA3*, *CDC20-AID::KanMX* |
| F1940 | MATa, *ura3-1::ADH1-OsTIR1-9Myc::URA3*, *pIPL1::TRP1::pMET3-UB-DHFR-IPL1-3HA::HIS3MX6*, *pURA3::tetR::GFP::LEU2*, *cenIV::tetOx448::URA3*, *CDC20-AID::KanMX* |
| F1942 | MATa, *ura3-1::ADH1-OsTIR1-9Myc::URA3*, *pIPL1::TRP1::pMET3-UB-DHFR-IPL1-3HA::HIS3MX6*, *pURA3::tetR::GFP::LEU2*, *cenIV::tetOx448::URA3*, *CDC20-AID::KanMX*, *mad1::HIS3MX6* |
| F1956 | MATa, *ipl1-as5::HIS3::KanMX6*, *pURA3::tetR::GFP::LEU2*, *cenIV::tetOx448::URA3*, *mad1::HIS3MX6* |
| F2414 | MATa, *ipl1-321*, *mad1::HIS3MX6* |
| F2493 | MATa, *ipl1-321*, *mad1::HIS3MX6*, *pURA3::tetR::GFP::LEU2*, *cenIV::tetOx448::URA3* |
